# Direct Comparison of Two Commonly Used Prenatal Valproate Doses Identifies Different Sex- and Age-Related Neurobehavioral Outcomes Across Development

**DOI:** 10.3390/neurosci7050112

**Published:** 2026-10-19

**Authors:** Maria Becker, Denis Gorobets, Boniface Ezenwata Echefu, Akiva Juroviesky, Anna Beylis, Aviv Sela, Asher Ornoy

**Affiliations:** 1 Department of Morphological Sciences and Teratology, Adelson School of Medicine, Ariel University, Ariel 40700, Israelbonifacee@ariel.ac.il (B.E.E.); akjuro@protonmail.com (A.J.); aviv.sela@mail.huji.ac.il (A.S.); ashero@ariel.ac.il (A.O.); 2 Hadassah Medical School, Hebrew University, Jerusalem 91010, Israel; asher.ornoy@mail.huji.ac.il

**Keywords:** prenatal, valproate, ASD model, dose–effect, sex differences, neurodevelopment, behavioral changes

## Abstract

Valproic acid (VPA) is a widely used anticonvulsant and mood stabilizer and a known teratogen associated with neural tube defects and neurodevelopmental disorders, including autism spectrum disorder (ASD). Prenatal VPA exposure is widely used to model ASD-like behaviors in rodents; however, direct comparisons between commonly used doses are lacking. We compared two widely used VPA doses (300 and 600 mg/kg) administered to pregnant mice on embryonic day 12.5 (E12.5). Male and female offspring were evaluated longitudinally for early neurological development, ultrasonic vocalizations, locomotor activity, anxiety-related behavior, sociability, and spontaneous alternation. Early development was largely preserved, although VPA300 delayed cliff aversion acquisition. Total ultrasonic vocalization production was unaffected, while selected call types were altered. Later behavioral effects differed across dose, sex, and age. In males, locomotor activity and center exploration were preserved, sociability was reduced by both VPA doses, and spontaneous alternation was reduced by VPA300 at postnatal day 70 (PND70). In females, locomotor activity was reduced by VPA600 at PND30 and VPA300 at PND70, center exploration was reduced by VPA600, and sociability was reduced by VPA300 at both ages and VPA600 at PND70. VPA600 was also associated with greater embryonic mortality. These findings show that the two VPA doses were associated with differing patterns of developmental and behavioral outcomes, without a consistent increase in severity at the higher dose.

## 1. Introduction

Valproic acid (VPA), an anticonvulsant and mood stabilizer, is one of the more potent teratogens associated with the etiology of Neural Tube Defects (NTDs) in humans. When taken during early pregnancy it increases the risk of NTDs 10- to 20-fold, to 1–2%, and the general risk of congenital malformations to 8–10% [1]. VPA use during pregnancy is also associated with a high rate of spontaneous abortions, reduced neonatal weight, and various brain malformations that can lead to severe neurodevelopmental problems [2,3]. Although VPA is a causative factor for only a limited number of NTDs in humans, it serves as a useful model for studying both NTD and Autism Spectrum Disorder (ASD) etiology, pathogenesis and prevention [2,4]. As a histone deacetylase (HDAC) inhibitor, VPA modulates neurotransmission and regulates gene expression by epigenetic chromatin remodeling [5,6,7]. Prenatal exposure to VPA causes transient hyperacetylation of H3 and H4 histones in the embryonic mouse brain [8], and the changes can be epigenetically transmitted, at least in rodents, to the second and even third generations [9].

Prenatal VPA exposure in mice shows that the neurobehavioral consequences are sensitive to sex and developmental timing. The critical windows of VPA administration in pregnancy to produce NTD (exencephaly) in mice are 8 and 9 gestational days and to produce offspring with ASD-like phenotypes is embryonic day 12.5 (E12.5), after neural tube closure [10,11,12]. Even a single dose of VPA resulted in most ASD-like symptoms [10,13,14] and anxiety, a common comorbid sign in autistic patients [15]. Kataoka et al. demonstrated that prenatal 500 mg/kg VPA on E12.5, but not at E9 or E14.5, induces autism-like behaviors in mice [8]. VPA exposure at E9 resulted in a high rate of malformed pups (30–40%), but the surviving normal pups did not exhibit behavioral abnormalities or autism-like traits. Moreover, VPA exposure at E14.5 did not result in behavioral abnormalities, histological changes, or neuronal loss, indicating that this stage is less sensitive to VPA-induced effects compared to E12.5 [8].

Most reproducible behavioral alterations across mouse strains appear to occur within a dosing range of approximately 300–600 mg/kg. A single prenatal VPA dose of 600 mg/kg administered around E12.5 is one of the most widely used protocols in rodent ASD models. This regimen consistently produces ASD-like behavioral phenotypes, including reduced sociability in three-chamber tests, increased repetitive grooming, anxiety-like behavior in the open field or elevated plus maze, and impairments in hippocampal-dependent memory tasks [8,16,17,18,19,20].

Intermediate doses of VPA (500 mg/kg) also produce ASD-like phenotypes with prominent reduction in dendritic spine density in the prefrontal cortex and hippocampus [21,22]. Lower doses of VPA (300–400 mg/kg) administered at GD12.5 have been less frequently studied but appear to produce deficits in social interaction behavior and social memory impairment [23]. Studies using 400 mg/kg in Swiss or ICR strains demonstrated alterations in repetitive behavior and anxiety-like responses, although the magnitude of these effects varied among strains [24].

Higher doses have also been examined under different experimental conditions. For example, 800 mg/kg VPA mixed with peanut butter to facilitate oral consumption, administered on E11, resulted in early developmental delays, impaired olfactory discrimination, and altered social behavior, accompanied by molecular changes in BDNF and NMDA receptor subunits [25].

The available reviews of rodent VPA models suggest that different prenatal doses produce different behavioral and molecular patterns, not necessarily graded by severity [10,13,14,26]. Higher doses (600–800 mg/kg) generally produce more consistent phenotypes, whereas lower and intermediate doses (300–500 mg/kg) often result in selective or sex-dependent alterations. Although both 300 and 600 mg/kg VPA are widely used in mouse models, we were unable to identify studies directly comparing these doses within the same strain and experimental framework.

In addition to dose, sex also appears to influence VPA-induced neurobehavioral outcomes. ASD is diagnosed approximately three to four times more frequently in males than females [27,28], but sex-specific effects in rodent VPA models remain inconsistent. Some studies report greater vulnerability in males, whereas others describe comparable or even stronger effects in females [17,29,30]. Behavioral abnormalities occur in offspring of both sexes, including reduced sociability, anxiety-like behavior, and repetitive movements. However, social deficits are typically more pronounced in males, whereas anxiety and repetitive phenotypes occur in both sexes [8,30]. These inconsistencies may partly reflect differences in VPA dose, mouse strain, behavioral paradigms, and the developmental stage at assessment.

Mouse strains used to induce ASD-like behavior by VPA also varied among reported studies. In outbred ICR mice, ASD-like behavioral alterations are observed in both sexes following prenatal VPA exposure, including after 300 mg/kg at E12.5 [17,28]. In contrast, many studies using inbred C57BL/6 mice employ doses around 600 mg/kg to obtain robust behavioral phenotypes, often accompanied by greater embryonic loss [19,20,31].

Thus, it remains unclear whether increasing VPA dose simply increases behavioral severity or instead is associated with different age- and sex-dependent developmental and behavioral outcome patterns. To address this question, we directly compared two commonly used prenatal VPA regimens (300 and 600 mg/kg) administered at E12.5 in ICR mice. We aimed to determine whether increasing VPA dose results in a simple increase in behavioral severity or is associated with different neurodevelopmental and behavioral outcome patterns across developmental stages and between sexes. Male and female offspring were evaluated longitudinally from early postnatal development through adulthood using measures of neurological maturation, ultrasonic vocalizations, open-field locomotion and center exploration, sociability, and spatial exploration in the Y-maze.

## 2. Materials and Methods

**Animals:** All animal experiments were approved by the Institutional Animal Care and Use Committee of Ariel University, Israel (approval # AU-IL-2309-112 and AU-IL2511-115), and were conducted in accordance with institutional guidelines and the NIH Guide for the Care and Use of Laboratory Animals for the humane use of animals in research. The animals were housed on a 12 h light/dark cycle, at 23  ±  2 °C ambient temperature, and had free access to standard laboratory food and water throughout testing.

**Breeding:** Adult male and female ICR mice were obtained at 7–8 weeks of age from Harlan (Israel) and randomly assigned to breeding pairs. All male mice used for breeding were approximately the same age as the females. The breeding pairs were monitored early in the morning for the presence of vaginal plugs, which is designated gestational day (GD) 0.5. Dams with detected vaginal plugs were weighed starting on GD7. Progressive increase in body weight was taken as confirmation of pregnancy.

**Embryonic/fetal mortality rate/resorptions.** Fetuses from 6 dams exposed to saline, 6 dams exposed to VPA (300 mg/kg, (Cat# P4543, Merck, Israel)) and 5 dams exposed to VPA (600 mg/kg) were evaluated at E16.0 by counting the number of viable and non-viable fetuses per litter.

**Figure 1:**
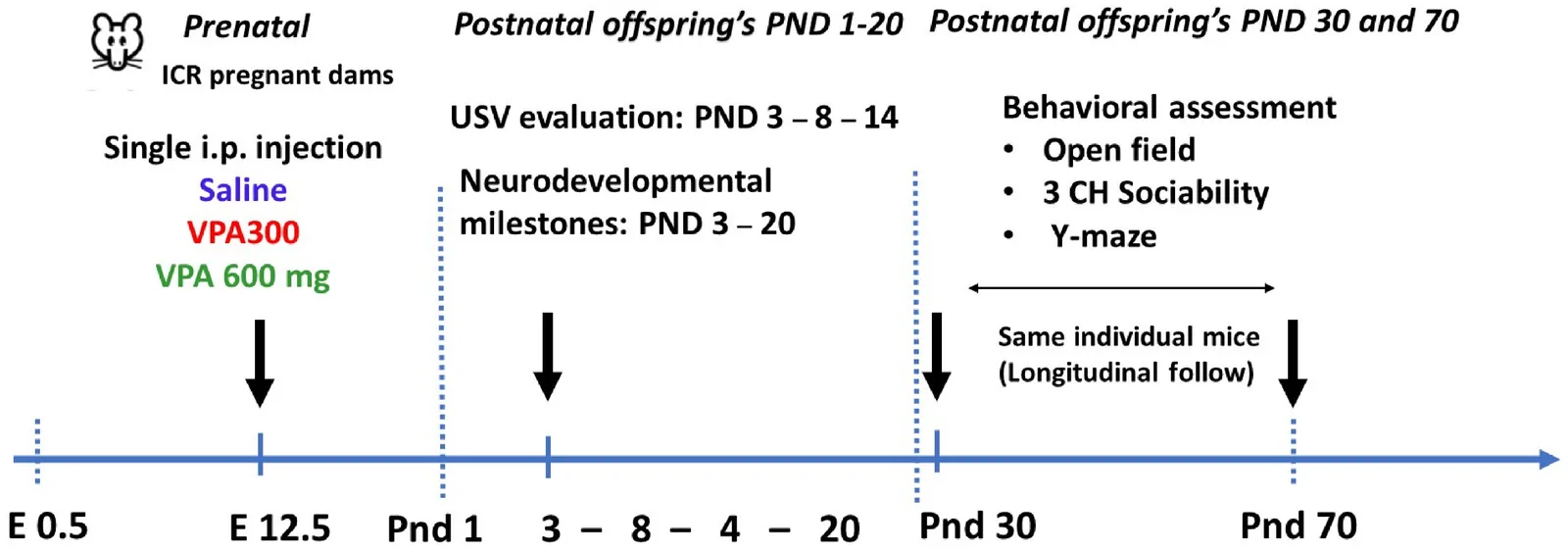
Experimental flow chart.

Figure 1 illustrates the overall experimental design of this study.

On E12.5, pregnant females (n = 32) were randomly divided into three experimental groups and received a single subcutaneous injection of 300 mg/kg or 600 mg/kg VPA (Cat# P4543, Merck, Israel) dissolved in saline. Control females received an equal volume of saline only. The day of birth was recorded as postnatal day (PND) 0. Each dam was kept in a single cage with their offspring until weaning. Offspring were marked with a non-toxic identification foot tattoo (Ketchum MFG, New York, NY, USA) and returned to the litter. Dams and their litters were housed together until weaning on PND21, when juvenile mice were weighed, ear-notched for permanent identification, and housed in same-sex-littermate groups.

**Neurological and behavioral development:** Mouse neurodevelopmental and behavioral patterns, particularly those related to autistic core symptoms, were evaluated from postnatal day (PND) 3 to PND70 using a battery of neurobehavioral tests. Milestone and USV assessments included partially overlapping sets of offspring and litters. The tests were conducted in the following order: neonatal ultrasonic vocalizations (PND3–14), neurodevelopmental milestones (PND3–20), open-field testing (locomotor activity and anxiety-like behavior), Y-maze testing (spatial exploration; PND30 and PND70), and the three-chamber social interaction test (PND31 and PND71). Body weight was measured every two days from PND3 to PND20 to monitor postnatal growth. Blinding to the prenatal treatment group was not implemented during the experimental procedures. Early developmental milestones were assessed manually using standardized protocols and predefined response criteria. USV recordings were processed and classified using the automated VocalMat machine-learning approach described by Fonseca et al. [32] with identical analytical settings applied to all recordings. Later behavioral measures were obtained from automated Noldus XT Ethovision14 video-tracking analysis (Noldus Information Technology, Wageningen, The Netherlands), thereby reducing the potential influence of observer bias on these outcomes.

No formal a priori power calculation was performed. The sample size was determined based on previous studies from our laboratory using the same VPA mouse model. Specifically, group sizes were selected according to earlier investigations that detected VPA-related behavioral alterations using comparable numbers of animals in behavioral experiments and studies that identified VPA-associated molecular changes using similar numbers of independent litters in a prenatal exposure design. The study was designed to examine multiple behavioral, developmental and molecular outcomes rather than a single predefined primary endpoint [11,33].

Offspring were obtained from multiple litters within each prenatal treatment group. Animals and litters substantially overlapped across developmental and behavioral assessments, although the exact number of offspring and contributing litter varied by endpoint. Developmental milestone assessments included offspring from 10 saline (28M + 34F), 8 VPA300 (34M + 41F), and 9 VPA600 (27M + 53F) litters, whereas adult behavioral assessments included offspring from up to 9 saline (39M + 29F), 4 VPA300 (14M + 17F), and 4 VPA600 (13M + 23F) litters. The same animals were assessed longitudinally at PND30 and PND70. Litter was included as a random effect in the statistical analyses to account for clustering of offspring within dams [34]. A schematic overview of cohort allocation is provided in Supplementary Figure S1, and the exact numbers of animals and litters contributing to each endpoint are reported in Supplementary Table S1.

**Ultrasonic vocalization (USV) recording and analysis:** USV recording and analysis were performed as described previously [35]. USVs were recorded on postnatal days (PND) 3, 8, and 14. Pups were separated from the dam for 30 min while remaining together with their littermates. Each pup was then individually placed in a rectangular plastic chamber (11 × 6 cm; wall height, 5 cm) located within a sound-insulating Styrofoam box and recorded for 3 min. USVs were recorded using an Ultravox XT2.0 microphone (Noldus Information Technology, The Netherlands) positioned 10 cm above the pup at a sampling rate of 250 kHz. After recording, pups were returned to the home cage with the dam. Recordings were filtered using a 20-kHz lower cutoff to reduce background noise and exported as WAV files.

Recorded USVs were evaluated using the VocalMat machine-learning approach described by Fonseca et al. [32], and the total number of calls, call types, and acoustic parameters were extracted. USVs were classified into 11 categories using the classification implemented in VocalMat, based on categories adapted from Grimsley et al. [36]: short, flat, upward frequency-modulated, downward frequency-modulated, complex, one-frequency step-up, one-frequency step-down, chevron, reverse chevron, multiple-frequency-step, and two-frequency-step calls.

**Neurodevelopmental milestones:**

Several neurodevelopmental milestones were evaluated in the offspring from PND3 to PND20 as outlined below. For each neurodevelopmental milestone, latency to perform the response was manually recorded during a maximum observation period of 30 s, according to the Fox and Heyser protocols [37,38]. Responses completed in <30 s were considered successful and assigned a score of 1, whereas responses requiring ≥30 s were considered failed and assigned a score of 0. Acquisition Day was defined as the first postnatal day on which the response criterion was achieved.

1. Surface righting reflex was measured as the time in seconds until the mouse pup turned back onto all four paws when placed on its back. The maximum latency to turn back was set at 30 s. A response time of ≥30 s was scored as 0 (failed), whereas a response time of <30 s was scored as 1 (successful).
2. Cliff aversion was measured as the time in seconds for pups positioned with their forepaws and snout over the edge of a shelf to turn and begin to crawl away from the edge. As in the surface righting test, a response time of ≥30 s was scored as 0 (failed), whereas a response time of <30 s was scored as 1 (successful).
3. Negative geotaxis was measured as the time in seconds required for a pup placed on an inclined plane (45°) to turn 180° and face upward. This test assesses proprioception of the pups. As in the surface righting test, a response time of ≥30 s was scored as 0 (failed), whereas a response time of <30 s was scored as 1 (successful).
4. Eye opening was defined as the first day on which both eyes were open.

**Behavioral tests:**

For behavioral assessments performed at PND30 and PND70, the same individual offspring were followed longitudinally and tested at both ages. Each animal was assigned a unique identification number and linked to its dam/litter, treatment group, and sex.

**Open field (OF) test.** The OF test is used to assess locomotor and exploratory activity and repetitive behavior [39]. Each mouse is placed individually in the center of the apparatus and evaluated for 10 min. Distance moved, number of entries and time spent in the arena center and outer zone were recorded using the Noldus XT video-tracking system and were analyzed to estimate exploration of a novel environment. Locomotor activity was assessed by distance moved. Exploration of the central and peripheral zones was assessed by time spent and entries into each zone. Time spent in the center is commonly used as an index of anxiety-related behavior and exploratory activity.

**Y-Maze:** The Y-maze spontaneous alternation test has been used to assess both repetitive behavior and spatial working memory [16]. The Y-maze was constructed of Plexiglas with three identical arms (42-cm long, 12-cm tall walls, illumination 30 lx). Each mouse was placed at the end of one arm and allowed to freely explore the maze for 6 min. The sequence of arm entries was recorded using the Noldus XT video-tracking system. An arm entry was scored when all four paws entered an arm. Spontaneous alternation was defined as consecutive entries into all three arms without repetition and was calculated as the percentage of alternations using the following formula:

Spontaneous alternation (%) = (Number of alternations/(Total arm entries − 2)) × 100.

Reduced spontaneous alternation is commonly interpreted as reflecting altered spatial working memory, although performance may also be influenced by exploratory behavior and other factors.

**Three-chamber social interaction test:** Crawley’s standard three-chamber social interaction test was used to evaluate social interaction [11]. Testing was carried out in two sessions in a three-chambered box with openings between the chambers. During all sessions, exploration was tracked using the Noldus XT video-tracking system. After a 5-min habituation session, an unfamiliar mouse (Stranger 1) was enclosed under a wire cup in one side chamber, while an identical empty wire cup was placed in the opposite chamber. Time spent exploring each stimulus was recorded. The sociability index is reflected in the preference for the social stimulus over the empty chamber. It was calculated by dividing the time spent in the social (stranger) vs. non-social (empty) zone by the total time spent in both, expressed as tStranger/(tStranger + tEmpty) × 100. Increased time spent exploring unfamiliar “stranger” age- and sex-matched mice indicates a high degree of sociability in the test animal.

**Statistical analysis:** Statistical analyses were performed using GraphPad Prism 11 (GraphPad Software, Boston, MA USA) and IBM SPSS Statistics 31.0.1.0(49) (IBM, Armonk, NY, USA). Graphical presentation of the data was performed using GraphPad Prism 11. Statistical significance was set at *p* < 0.05. Unless otherwise specified, mixed-effects models were fitted by restricted maximum likelihood (REML), with Satterthwaite approximation for degrees of freedom. Litter was incorporated as a random effect to account for clustering of offspring within litter. For repeated-measures analyses, the covariance structure was specified according to the structure of the repeated observations, using compound symmetry (CS), first-order autoregressive [AR(1)], or unstructured (UN) covariance as appropriate.

For embryotoxicity, fetal mortality was calculated as the percentage of dead or resorbed embryos within each litter, with the litter used as the experimental unit. Differences among prenatal treatment groups were analyzed using the Kruskal–Wallis test followed by Dunn’s multiple-comparisons test in GraphPad Prism.

For developmental milestones, acquisition day was defined as the first postnatal day on which the criterion response was observed according to established protocols [37,38]. Acquisition day was analyzed using linear mixed-effects models with treatment, sex, and the treatment × sex interaction as fixed effects and litter as a random effect. Cumulative acquisition curves showing the percentage of offspring that had acquired each response by postnatal day were used for graphical presentation. For cliff aversion, time to acquisition was additionally analyzed using Complex Samples Cox proportional-hazards regression, with litter specified as the clustering unit; effect estimates are reported as hazard ratios (HRs) with 95% confidence intervals [40]. These analyses were performed in IBM SPSS Statistics.

For USV analyses, total call number and the numbers of individual syllable types were analyzed using linear mixed-effects models with treatment, sex, age, and their interactions as fixed effects; litter as a random effect; and repeated observations across age modeled within individual offspring. Models were fitted by restricted maximum likelihood (REML), with Satterthwaite approximation for degrees of freedom. Significant effects were followed by Bonferroni-adjusted pairwise comparisons. To account for multiple testing across the individual syllable-type analyses, *p*-values for the corresponding interaction effects were additionally adjusted using the Benjamini–Hochberg false discovery rate (FDR) procedure [41].

For longitudinal behavioral outcomes assessed at PND30 and PND70, linear mixed-effects models were used to evaluate the effects of treatment, sex, age, and their interactions while accounting for the hierarchical and repeated-measures structure of the data. Litter was included as a random effect, and repeated observations across age were modeled within individual offspring. Models were fitted by REML, with Satterthwaite approximation for degrees of freedom. Significant effects were followed by Bonferroni-adjusted pairwise comparisons. These analyses were performed using IBM SPSS Statistics.

Multiplicity adjustment was performed within individual analytical models and behavioral domains rather than across the entire set of investigated endpoints. Bonferroni-adjusted pairwise comparisons were applied within individual mixed-effects models, and FDR correction was additionally applied to USV subtype analyses. Accordingly, the study should be regarded as exploratory rather than confirmatory, and findings with *p*-values close to the significance threshold should be interpreted cautiously.

## 3. Results

### 3.1. Effect of Prenatal VPA 300 mg/kg and 600 mg/kg Treatment on Embryonic Mortality Rate

Prenatal VPA exposure was associated with increased embryonic mortality, most pronounced at the higher dose. Fetuses from 6 dams exposed to saline, VPA (300 mg/kg) and 5 dams exposed to VPA (600 mg/kg) were evaluated at E16.0 by counting the number of viable and non-viable fetuses per litter and embryonic mortality was analyzed at the litter level, with each dam/litter considered an independent experimental unit. For each litter, mortality was calculated as the percentage of non-viable fetuses among the total number of fetuses. The pooled descriptive mortality rates were 0.95%, 8.5%, and 33.7% in the saline, VPA300, and VPA600 groups, respectively. Litter-level mortality differed significantly among treatment groups (Kruskal–Wallis H = 8.934, exact *p* = 0.006). Dunn’s multiple-comparisons test showed significantly higher mortality in the VPA600 group than in saline controls (adjusted *p* = 0.0091), whereas VPA300 did not differ significantly from saline (*p* = 0.8) or VPA600 (*p* = 0.16). Mean litter size was comparable among groups, indicating that the effect reflects increased embryonic death rather than altered fertility (Table 1).

### 3.2. Effect of Prenatal VPA 300 mg/kg and 600 mg/kg Treatment on Postnatal Weight Gain and Eye Opening in Male and Female Offspring in Comparison to Controls Treated with Saline

**Figure 2:**
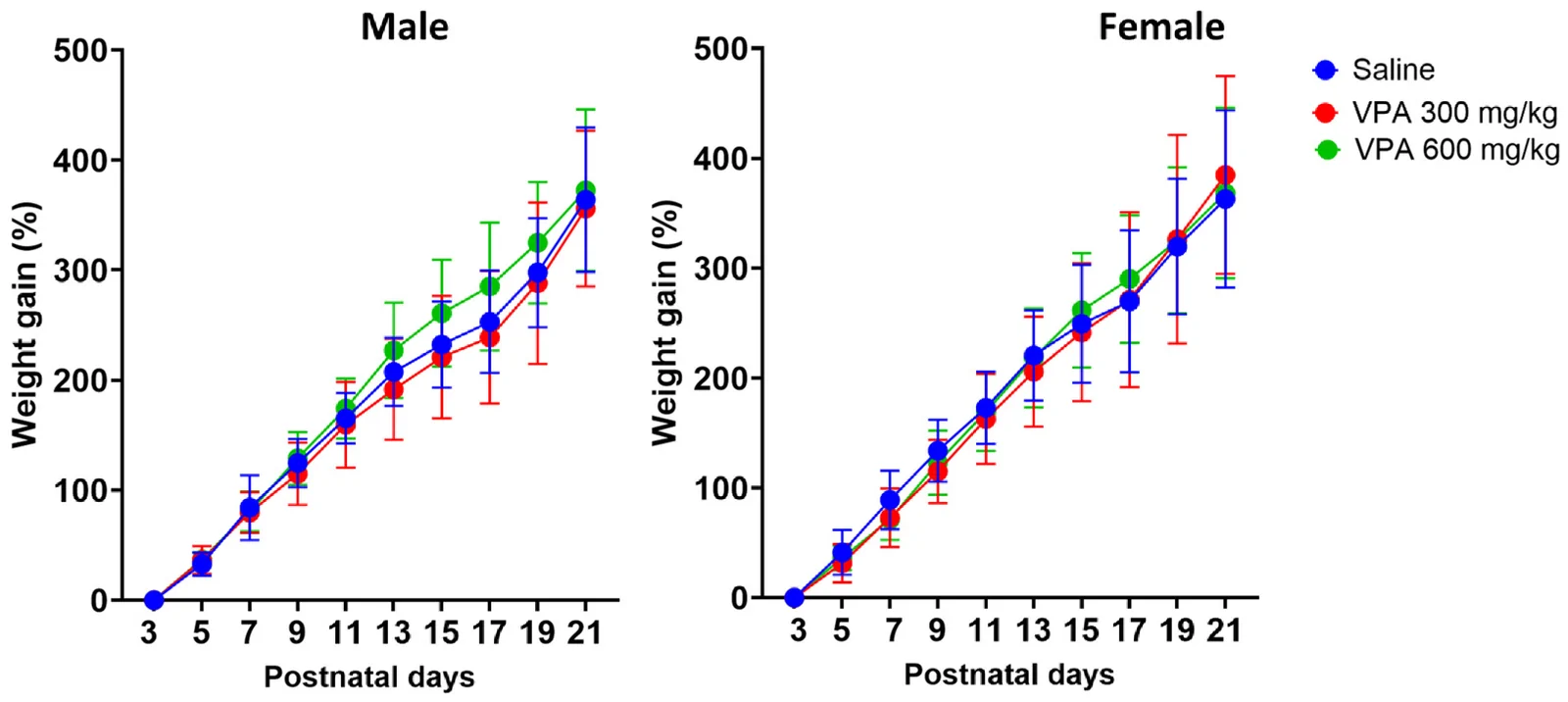
Postnatal weight gain in male and female pups following prenatal exposure to VPA. Body-weight gain from PND3 to PND21 in male and female offspring prenatally exposed to saline (blue), VPA 300 mg/kg (red), or VPA 600 mg/kg (green). Body weight increased progressively across postnatal development in all groups, with no overall treatment effect (F(2, 21.93) = 0.79, *p* = 0.468). Significant Treatment × Age (F(32, 2034.89) = 1.98, *p* < 0.001) and Treatment × Sex × Age interactions (F(32, 2034.17) = 1.63, *p* = 0.014) were detected. In males, body weight was greater in the VPA600 than in the VPA300 group at PND14, PND15, and PND17 (Bonferroni-adjusted *p* = 0.013, 0.040, and 0.033, respectively); no significant pairwise treatment differences were detected in females. Neither VPA300 nor VPA600 differed significantly from saline at any time point after correction for multiple comparisons. Data are presented as mean ± SEM.

We assessed the weight gain in both male and female offspring from PND3 to 21 (Figure 2). Body weight increased progressively across all groups, with no overall treatment effect (F(2, 21.93) = 0.79, *p* = 0.468). However, significant Treatment × Age (F(32, 2034.89) = 1.98, *p* < 0.001) and Treatment × Sex × Age interactions [F(32, 2034.17) = 1.63, *p* = 0.014] indicated that treatment effects were not entirely uniform across ages and sexes. In males, greater weight gain was observed in the VPA600 than in the VPA300 group at PND14, PND15, and PND17 (Bonferroni-adjusted *p* = 0.013, 0.040, and 0.033, respectively). No significant pairwise treatment differences were detected in females. Importantly, neither VPA300 nor VPA600 differed significantly from saline at any time point after correction for multiple comparisons, indicating no evidence of impaired postnatal growth following prenatal VPA exposure.

Eye-opening timing was also unaffected by prenatal VPA exposure. Analysis of acquisition day revealed no significant effect of treatment [F(2, 14.56) = 0.28, *p* = 0.758], sex [F(1, 81.98) = 2.22, *p* = 0.140], or Treatment × Sex interaction [F(2, 81.90) = 0.27, *p* = 0.763]. No significant Bonferroni-adjusted pairwise differences were detected among treatment groups, indicating no evidence that either VPA dose altered the timing of eye opening. Together, these findings indicate that postnatal growth and eye-opening timing were largely preserved following prenatal VPA exposure.

### 3.3. Effects of Prenatal VPA 300 mg/kg and 600 mg/kg Treatment on Ultrasonic Vocalization Quantity and Call-Type Composition

In male offspring, the number of vocalizations in the 3-min recorded sonogram followed the expected developmental trajectory, with a peak at PND8 and decline by PND14.

**Figure 3:**
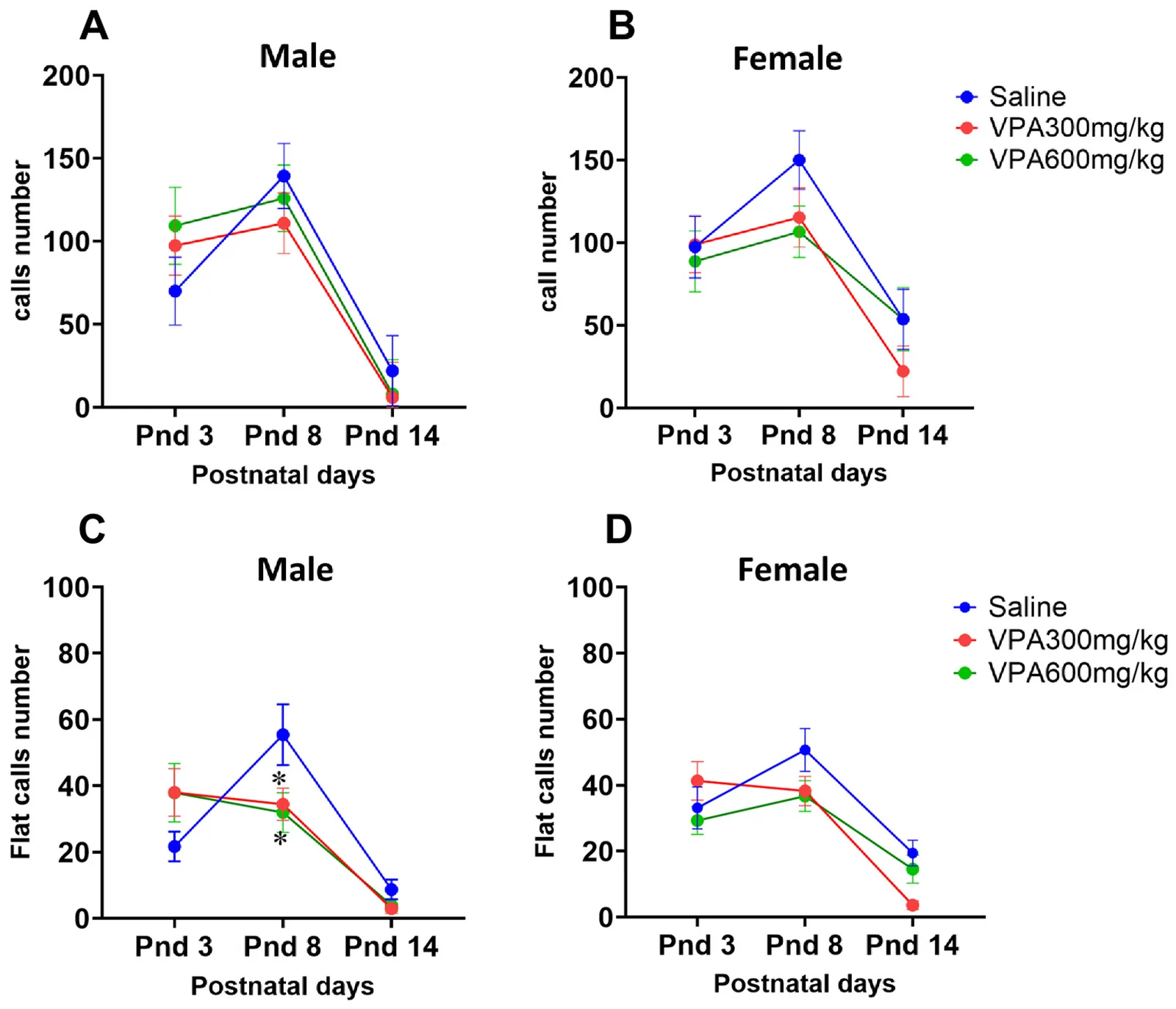
Total ultrasonic vocalization (USV) production and Flat calls in male and female offspring following prenatal VPA exposure. Effects of prenatal VPA exposure on ultrasonic vocalization production and Flat call number at PND3, PND8, and PND14. Ultrasonic vocalizations were assessed during 3-min recording sessions at PND3, PND8, and PND14 in male and female offspring prenatally exposed to saline (blue), VPA 300 mg/kg (red), or VPA 600 mg/kg (green). (**A**,**B**) show total USV call number; (**C**,**D**) show the number of Flat calls. Total USV production showed a marked age-related pattern in both sexes, with increased call numbers at PND8 followed by a decline at PND14, but no significant treatment differences at any age. Flat calls also varied with age. In males, both VPA300 (*p* = 0.049) and VPA600 (*p* = 0.03) groups produced fewer Flat calls than saline controls at PND8, whereas no significant treatment differences were detected in females. Data are presented as mean ± SEM. Asterisks indicate significant pairwise differences versus saline (* *p* < 0.05).

Total USV emitted calls showed a marked developmental change across postnatal ages (Figure 3A,B). Linear mixed-effects analysis, with litter included as a random effect and age treated as a repeated measure within offspring, showed a significant effect of age (F(2, 309.65) = 64.67, *p* < 0.001) but no significant effects of treatment (F(2, 18.49) = 0.46, *p* = 0.640) or sex (F(1, 187.15) = 1.05, *p* = 0.306). The Treatment × Age (*p* = 0.086), Treatment × Sex (*p* = 0.433), Sex × Age (*p* = 0.337), and Treatment × Sex × Age (*p* = 0.408) interactions were also not significant. Bonferroni-adjusted comparisons showed no significant treatment differences in total call number at PND3, PND8, or PND14 in either sex. Thus, the developmental trajectory of total USV production was largely preserved following prenatal VPA exposure.

Analysis of individual syllable types indicated that treatment-related differences were restricted to selected components of the USV repertoire. Flat calls showed a significant Treatment × Age interaction (F(4, 316.42) = 5.35, *p* < 0.001), which remained significant after FDR correction (q ≤ 0.011, Figure 3C,D). At PND8, males in both VPA groups produced fewer Flat calls than saline controls (VPA300, *p* = 0.049; VPA600, *p* = 0.029; Bonferroni-adjusted comparisons). No significant treatment differences were detected in females at this age, and the Treatment × Sex × Age interaction was not significant (*p* = 0.301). Several less frequent syllable types, including complex and reverse-chevron calls, also showed significant interactions in the initial analyses; however, because these call types occurred at very low frequencies and were produced by relatively few animals, these findings were not considered sufficiently robust for biological interpretation.

Together, the analyses of total USV production and syllable types suggest that prenatal VPA exposure did not produce a generalized suppression of vocalization but was associated with changes in specific components of the vocal repertoire. Total call number was not significantly affected by treatment, whereas analysis of syllable composition identified treatment-related variation in selected call types. Among these, the reduction in Flat calls at PND8 in VPA-exposed males represented the most clearly interpretable pattern. Although significant interactions were also detected for some less frequent syllable types, including complex and reverse-chevron calls, their low occurrence limits biological interpretation of these effects. Thus, the findings suggest that prenatal VPA exposure may influence the composition of the developing vocal repertoire without substantially reducing overall USV production.

### 3.4. Effects of Prenatal VPA 300 mg/kg and 600 mg/kg Exposure on Early Neurodevelopmental (Neurological) Milestones

**Figure 4:**
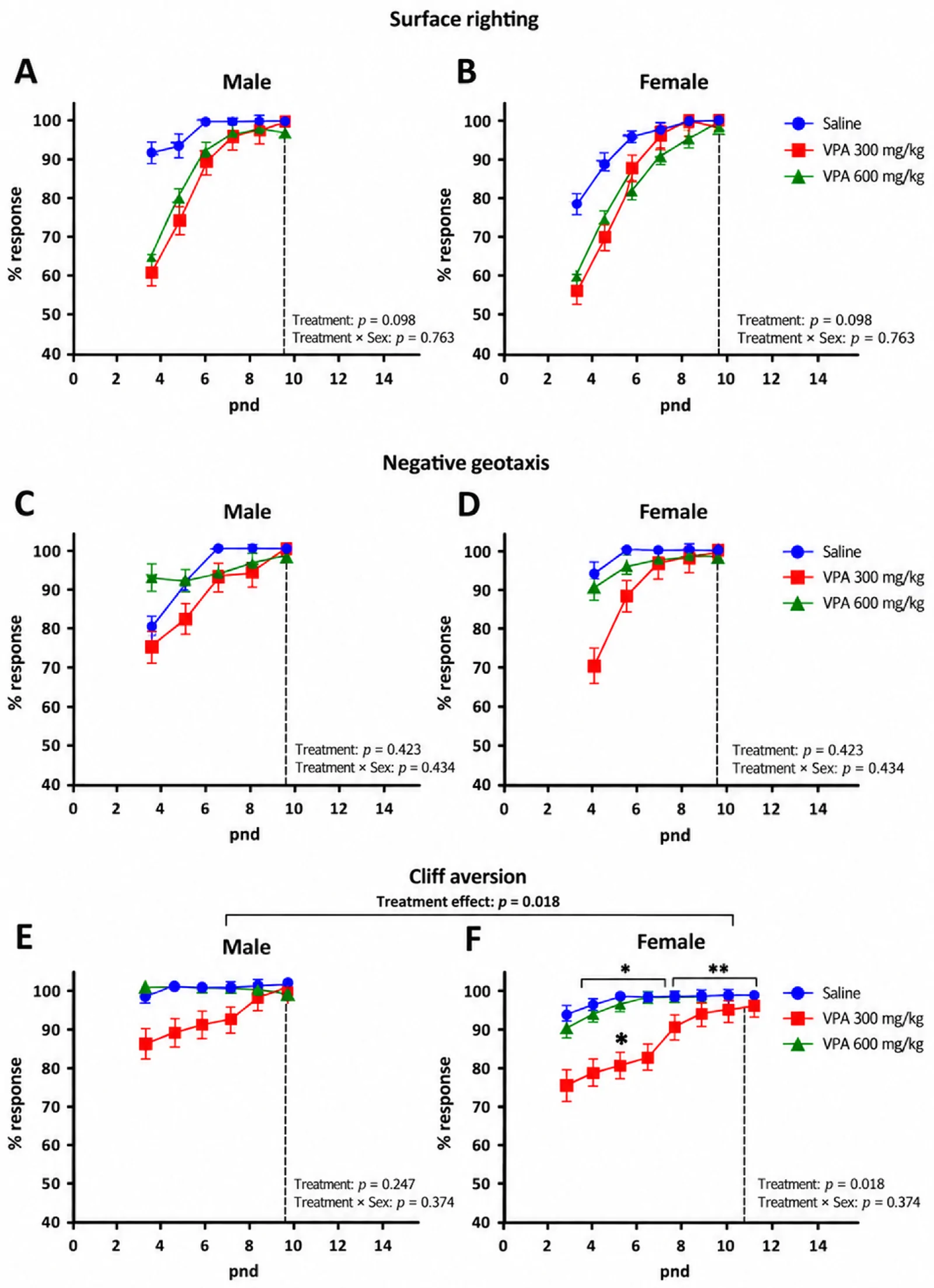
Cumulative percentage of offspring acquiring developmental milestones across postnatal days. Developmental acquisition curves of surface righting (**A**,**B**), negative geotaxis (**C**,**D**), and cliff aversion (**E**,**F**) represent the cumulative proportion of pups achieving each milestone across postnatal days. Curves were generated using nonlinear sigmoidal fitting for visualization. Data are shown separately for male (**A**,**C**,**E**) and female (**B**,**D**,**F**) offspring. Early differences in the cumulative acquisition trajectories were observed for surface righting, whereas negative geotaxis was largely preserved across groups. Cliff aversion showed the most pronounced separation between groups, particularly in VPA300-exposed offspring, followed by convergence toward near-complete acquisition. A significant treatment effect was detected for cliff aversion (*p* = 0.018), whereas treatment effects were not significant for surface righting (*p* = 0.098) or negative geotaxis (*p* = 0.423). Data are presented as individual values with mean ± SEM. Asterisks indicate significant pairwise differences (* *p* < 0.05, ** *p* < 0.01).

To evaluate the impact of prenatal VPA exposure on early sensorimotor maturation, we assessed the acquisition of three core neurodevelopmental milestones: surface righting (SR), negative geotaxis (NG) and cliff aversion (CA), in male and female offspring from postnatal day 4 to 15 (Figure 4).

Surface righting acquisition was largely preserved in both sexes, with all pups reaching maximal performance within a narrow postnatal window. Although the cumulative acquisition curves showed early differences between treatment groups, analysis of acquisition day using a linear mixed-effects model with litter included as a random effect showed no significant effect of treatment (F(2, 21.02) = 2.60, *p* = 0.098), sex (F(1, 198.26) = 0.49, *p* = 0.487), or Treatment × Sex interaction (F(2, 198.24) = 0.27, *p* = 0.763). Thus, overall surface righting acquisition did not differ significantly between treatment groups (Table 2). These results indicate largely preserved surface righting acquisition, despite transient differences in the early acquisition trajectories (Figure 4A,B).

Negative geotaxis showed some early variation in the cumulative acquisition curves, while overall acquisition was largely preserved in both sexes. Analysis of acquisition day showed no significant effect of treatment (F(2, 25.35) = 0.89, *p* = 0.423), sex (F(1, 189.20) = 1.89, *p* = 0.171), or Treatment × Sex interaction (F(2, 186.04) = 0.84, *p* = 0.434) after accounting for litter as a random effect. No significant pairwise treatment differences in acquisition day were detected. Thus, the observed differences in the cumulative curves were not accompanied by a significant overall alteration in negative geotaxis acquisition (Table 2; Figure 4C,D).

Cliff aversion showed the clearest effect of prenatal VPA exposure among the developmental milestones examined, although acquisition was ultimately preserved in nearly all offspring within the developmental assessment period. Analysis of acquisition day using a linear mixed-effects model with litter included as a random effect showed a significant effect of treatment (F(2, 22.15) = 4.86, *p* = 0.018), whereas the effect of sex (F(1, 206.98) = 2.23, *p* = 0.137) and the Treatment × Sex interaction (F(2, 207.79) = 0.99, *p* = 0.374) were not significant. Bonferroni-adjusted pairwise comparisons showed later acquisition in VPA300 females compared with both saline (*p* = 0.011) and VPA600 females (*p* = 0.009), whereas no significant pairwise treatment differences were detected in males. Thus, prenatal VPA exposure altered the timing of cliff-aversion acquisition, with later acquisition observed in VPA300 females compared with both saline and VPA600 females. However, the absence of a significant Treatment × Sex interaction does not support a statistically established sex-specific treatment effect (Figure 4E,F; Table 2).

Because one pup did not achieve cliff aversion by the end of the assessment period and therefore had no defined acquisition day for inclusion in the mixed-effects analysis, a complementary time-to-event analysis was performed to assess the robustness of this finding while retaining this animal as right-censored. A Complex Samples Cox regression accounting for litter clustering confirmed an overall effect of treatment on the rate of cliff-aversion acquisition (Wald F(2, 19) = 4.81, *p* = 0.020). Compared with saline, VPA300 offspring showed a lower acquisition hazard (HR = 0.611, 95% CI 0.422–0.885, *p* = 0.012), consistent with delayed acquisition, whereas VPA600 did not differ from saline (HR = 1.049, 95% CI 0.871–1.262, *p* = 0.600).

Together, the mixed-effects and time-to-event analyses indicate a delayed acquisition of cliff aversion following prenatal VPA300 exposure, while acquisition in the VPA600 group did not differ significantly from saline controls.

Together, these results indicate that prenatal VPA exposure produced limited alterations in early neurodevelopmental milestone acquisition, with a significant treatment effect observed for cliff aversion. Pairwise comparisons showed later cliff-aversion acquisition in VPA300 females compared with both saline and VPA600 females; however, the absence of a significant Treatment × Sex interaction does not support a sex-specific treatment effect. Surface righting and negative geotaxis were largely preserved, with no significant overall treatment effects on acquisition timing. Importantly, nearly all offspring ultimately acquired the developmental milestones within the assessment period, indicating altered timing of maturation rather than persistent impairment.

### 3.5. Effects of Prenatal VPA 300 mg/kg and 600 mg/kg Exposure on Behavioral Traits Measured at Postnatal Days 30 (Adolescence) and 70 (Adulthood)

#### 3.5.1. Locomotor Activity and Center Exploration Assessed by Open Field

**Figure 5:**
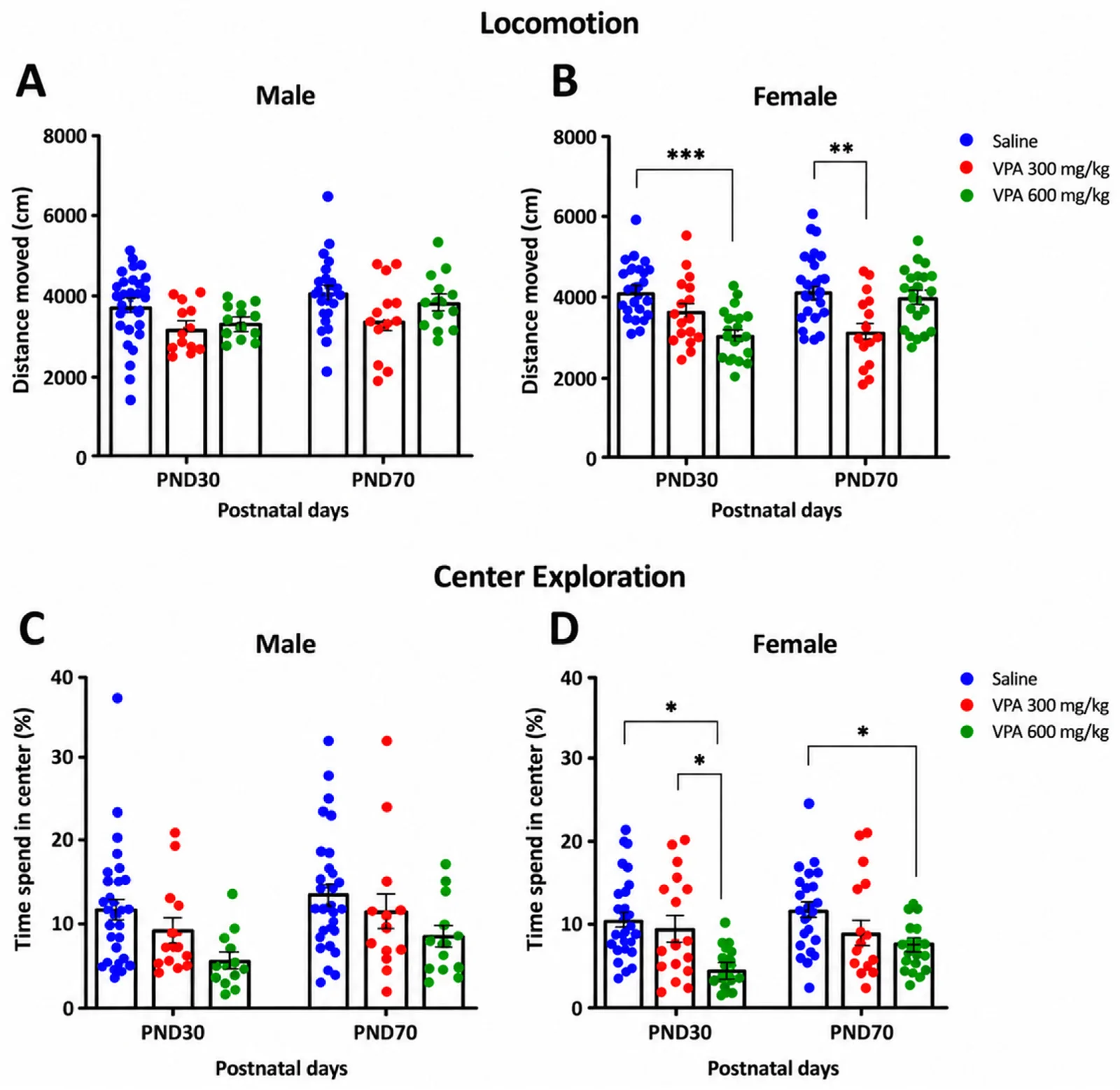
Effects of prenatal VPA exposure on locomotor activity and center exploration in the open-field test at PND30 and PND70. Open-field performance was assessed at PND30 and PND70 in male and female offspring prenatally exposed to saline (blue), VPA 300 mg/kg (red), or VPA 600 mg/kg (green). (**A**,**B**) show total distance moved (cm) as an index of locomotor activity; (**C**,**D**) show time spent in the center of the arena (%) as a measure of center exploration. In males, locomotor activity showed no significant treatment differences at either age, and center exploration was similarly preserved across treatment groups. In females, locomotor activity showed an age- and treatment-dependent pattern, with reduced distance moved in the VPA600 group at PND30 and in the VPA300 group at PND70. Center exploration was reduced in the VPA600 group at both PND30 and PND70; at PND30, VPA600 females also showed reduced center time compared with VPA300 females. Data are presented as individual values with mean ± SEM. Asterisks indicate significant pairwise differences (* *p* < 0.05, ** *p* < 0.01, *** *p* < 0.001).

Locomotor activity showed treatment-related changes that varied with age and sex. In males, distance moved was not significantly altered by either VPA dose at PND30 or PND70. In females, locomotor activity was reduced by VPA600 at PND30, whereas at PND70 the reduction was observed with VPA300 but not VPA600. Linear mixed-effects analysis of distance moved revealed significant main effects of treatment (F(2, 14.271) = 5.350, *p* = 0.018) and age (F(1, 130) = 13.255, *p* < 0.001), together with significant Treatment × Age (F(2, 130) = 5.326, *p* = 0.006) and Treatment × Sex × Age interactions (F(2, 130) = 3.211, *p* = 0.044). Pairwise comparisons showed no significant treatment differences in males at either PND30 or PND70 (Figure 5A). In females, distance moved was reduced in the VPA600 group compared with saline at PND30 (*p* = 0.001), whereas VPA300 did not differ from saline (Figure 5B). At PND70, distance moved was reduced in VPA300 females compared with saline (*p* = 0.005), whereas VPA600 did not differ from saline. The VPA300 and VPA600 groups did not differ significantly at PND70 (*p* = 0.065).

Center exploration showed age-related variation in the treatment effect. No significant treatment differences were detected in males at either age. Whereas in females VPA600 was associated with reduced center time at both PND30 and PND70. At PND30, VPA600 females also spent less time in the center than VPA300 females, whereas VPA300 did not differ from saline. VPA300 did not differ significantly from saline at either age. Mixed-effects analysis of time spent in the center revealed a significant effect of treatment (F(2, 15.050) = 3.971, *p* = 0.041) and a Treatment × Age interaction (F(2, 130) = 3.161, *p* = 0.046). The Treatment × Sex × Age interaction was not significant. Pairwise comparisons showed no significant treatment differences in males at either PND30 or PND70 (Figure 5C). In females, VPA600 offspring spent less time in the center than saline controls at both PND30 (*p* = 0.045) and PND70 (*p* = 0.026). At PND30, center time was also lower in VPA600 than in VPA300 females (*p* = 0.013), whereas VPA300 did not differ from saline. At PND70, VPA300 did not differ significantly from either saline or VPA600 (Figure 5D).

Thus, prenatal VPA exposure did not produce a uniform increase in behavioral effects with the higher dose. Locomotor activity was preserved in males, whereas females showed reduced activity with VPA600 at PND30 and VPA300 at PND70. Center exploration was also preserved in males, while VPA600 females showed reduced center time at both ages. At PND30, this reduction was accompanied by reduced locomotor activity, which limits its interpretation as an anxiety-related effect. At PND70, however, center time remained reduced without a significant change in locomotor activity. These findings indicate that the behavioral effects of the two VPA doses varied according to age and behavioral outcome, without a consistent increase in severity from 300 to 600 mg/kg.

#### 3.5.2. Sociability Assessed by Three-Chamber Test

**Figure 6:**
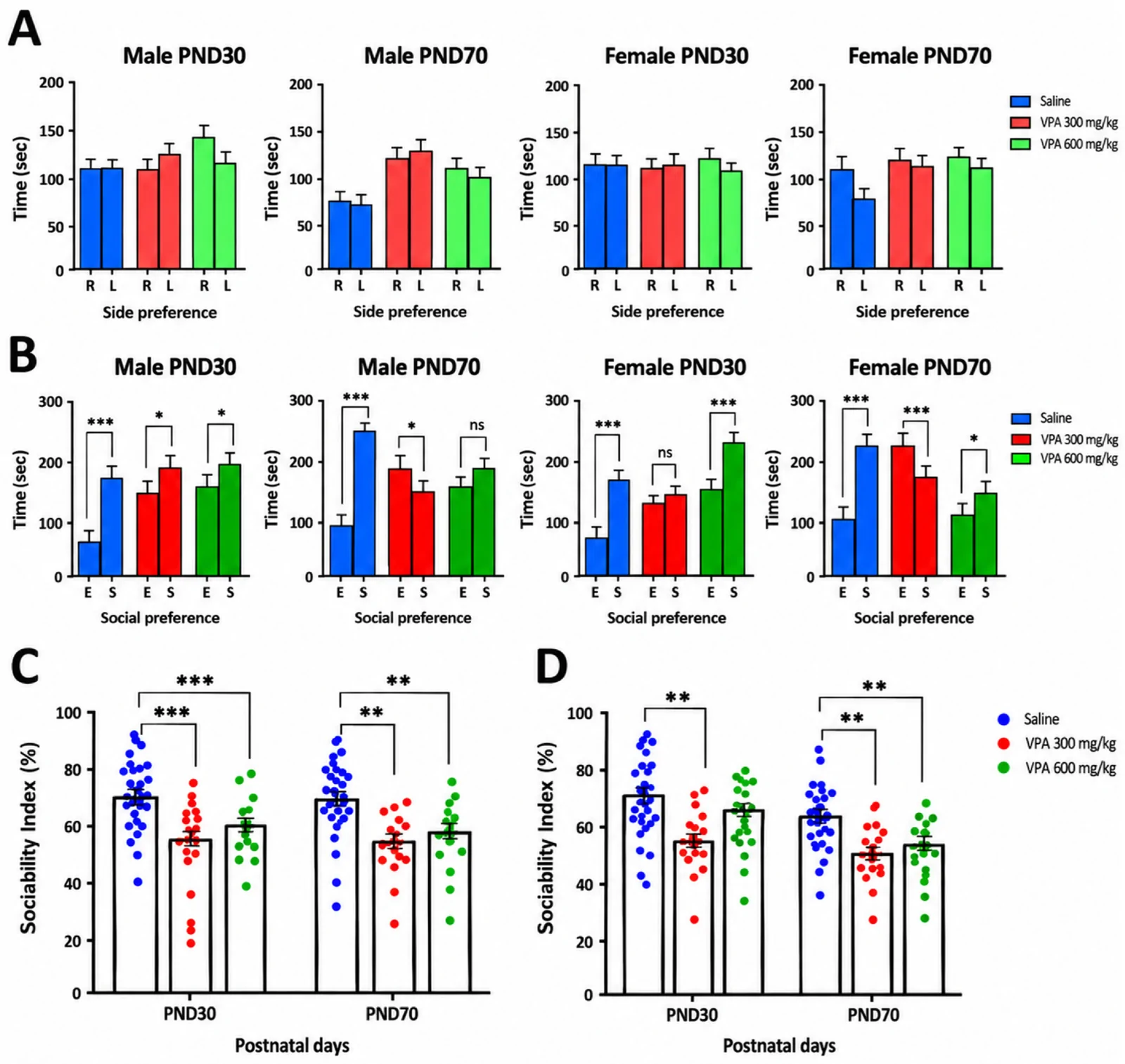
Effects of prenatal VPA exposure on social preference and sociability in the three-chamber test at PND30 and PND70. Three-chamber social behavior was assessed at PND30 and PND70 in male and female offspring prenatally exposed to saline (blue), VPA 300 mg/kg (red), or VPA 600 mg/kg (green). Panel (**A**) shows right (R) and left (L) chamber occupancy during habituation, with no significant initial side preference in any treatment group at either age. Panel (**B**) shows exploration time of the stranger (S) and empty (E) stimulus areas. Saline offspring showed greater exploration of the stranger at both ages and in both sexes. In VPA300 offspring, stranger preference was altered with age, with a reversal toward greater empty-stimulus exploration at PND70 in both sexes, whereas VPA600 offspring generally retained stranger preference, except for males at PND70. However, no significant direct difference between VPA300 and VPA600 was detected. Panels (**C**,**D**) show the sociability index in male and female offspring, respectively. In males, sociability was reduced in both VPA groups at PND30 and PND70. In females, sociability was reduced in the VPA300 group at PND30 and in both VPA groups at PND70. No significant direct VPA300–VPA600 difference in sociability was detected at either age. Data are presented as mean ± SEM in (**A**,**B**) and as individual values with mean ± SEM in (**C**,**D**). Asterisks indicate significant pairwise differences (* *p* < 0.05, ** *p* < 0.01, *** *p* < 0.001); ns, not significant.

Initial chamber-side preference during habituation was examined to exclude pre-existing side bias (Figure 6A). At PND30, there was no significant effect of side (F(1, 84) = 0.288, *p* = 0.593) and no Treatment × Side (F(2, 84) = 1.554, *p* = 0.217), Sex × Side (F(1, 84) < 0.001, *p* = 0.987), or Treatment × Sex × Side interaction (F(2, 84) = 0.452, *p* = 0.638). Similarly, at PND70, there was no significant effect of side (F(1, 84) = 0.377, *p* = 0.541), Treatment × Side (F(2, 84) = 1.123, *p* = 0.330), Sex × Side (F(1, 84) = 1.905, *p* = 0.171), or Treatment × Sex × Side interaction (F(2, 84) = 0.274, *p* = 0.761). Thus, subsequent differences in social exploration were not attributable to an initial preference for one side of the apparatus.

Analysis of stranger- and empty-cage exploration showed that saline offspring consistently preferred the stranger at both ages, whereas prenatal VPA exposure altered this pattern according to treatment and age. Loss or reversal of stranger preference was observed in the VPA300 group, particularly at PND70, whereas VPA600 offspring showed a more variable age-dependent pattern. Saline males and females spent significantly more time exploring the stranger than the empty cage at both PND30 and PND70 (all *p* < 0.001) (Figure 6B). VPA300 males retained stranger preference at PND30 (*p* = 0.032), but at PND70, they spent significantly less time exploring the stranger than the empty cage (*p* = 0.020). VPA300 females showed no significant preference at PND30 (*p* = 0.250) and spent less time exploring the stranger at PND70 (*p* = 0.001). VPA600 males preferred the stranger at PND30 (*p* = 0.031), whereas this comparison did not reach significance at PND70 (*p* = 0.065). VPA600 females retained significant stranger preference at both PND30 (*p* < 0.001) and PND70 (*p* = 0.034). Consistent with these age-dependent patterns, the Treatment × Age × Stimulus interaction was significant (F(2, 89.449) = 9.448, *p* < 0.001), whereas the Treatment × Sex × Age × Stimulus interaction was not significant (F(2, 89.499) = 1.063, *p* = 0.350).

The sociability index showed a broader reduction following prenatal VPA exposure. In males, sociability was reduced by both VPA doses at PND30 and PND70 (Figure 6C). In females, the reduction was observed with VPA300 at PND30 and with both VPA doses at PND70 (Figure 6D). Litter-adjusted mixed-effects analysis across PND30 and PND70 showed significant main effects of treatment (F(2, 14.478) = 23.884, *p* < 0.001) and age (F(1, 136) = 19.858, *p* < 0.001), whereas the main effect of sex was not significant (F(1, 123.724) = 0.177, *p* = 0.675). Significant Treatment × Sex (F(2, 123.942) = 3.192, *p* = 0.044) and Treatment × Age (F(2, 136) = 4.753, *p* = 0.010) interactions were detected, whereas the Treatment × Sex × Age interaction was not significant (F(2, 136) = 0.128, *p* = 0.880). In males, sociability was reduced relative to saline in both the VPA300 and VPA600 groups at PND30 (both *p* < 0.001) and PND70 (VPA300, *p* < 0.001; VPA600, *p* = 0.001), with no significant difference between the two VPA doses at either age. In females, sociability was reduced in VPA300 compared with saline at PND30 (*p* = 0.004), whereas VPA600 did not differ significantly from saline (*p* = 0.059). At PND70, both the VPA300 (*p* < 0.001) and VPA600 (*p* = 0.019) groups showed reduced sociability compared with saline, with no significant difference between the VPA groups (*p* = 0.065).

Thus, both stranger-versus-empty exploration and the sociability index showed that prenatal VPA exposure altered social behavior, with effects varying according to dose and age. Stranger-versus-empty exploration showed differing patterns across the two VPA groups, including altered preference in VPA300 offspring at PND70, without establishing a direct difference between the VPA doses. For the sociability index, both VPA doses showed an overall reduction relative to saline, with no significant overall difference between VPA300 and VPA600; significant Treatment × Sex and Treatment × Age interactions indicated that treatment effects varied according to sex and age.

#### 3.5.3. Y-Maze Spontaneous Alternation and Arm Entries

**Figure 7:**
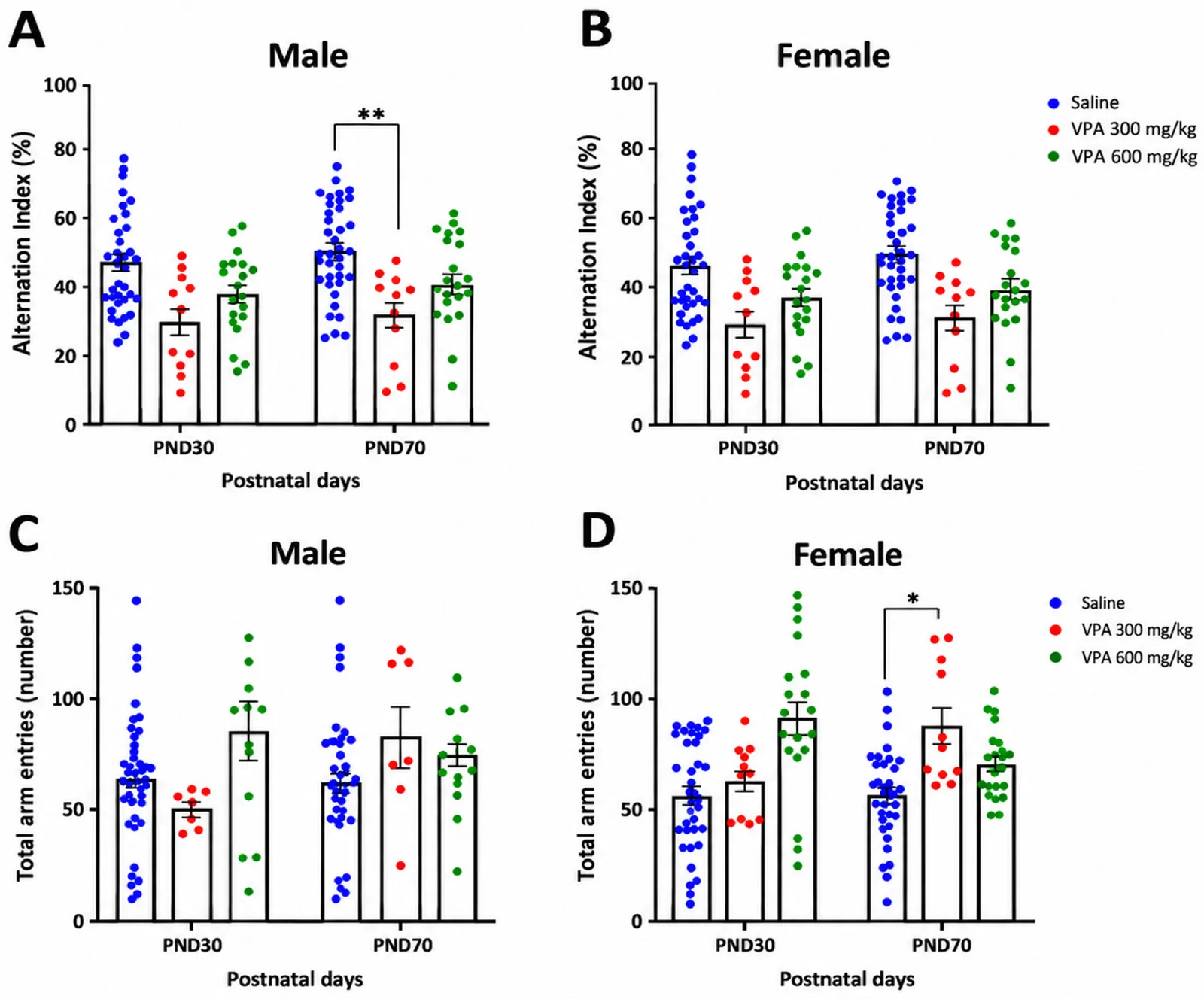
Prenatal VPA exposure alters Y-maze spontaneous alternation and total arm entries across development. Males (**A**) showed reduced spontaneous alternation in the VPA300 mg/kg group relative to saline at PND70, whereas no significant treatment differences were observed at PND30. In females (**B**), no individual treatment comparisons reached significance at either age after correction for multiple comparisons. Analysis of total arm entries showed no significant differences among treatment groups in males at either PND30 or PND70 (**C**). In females (**D**), no treatment differences were observed at PND30, whereas VPA300 mg/kg offspring showed increased total arm entries relative to saline at PND70. Data are presented as individual animals with mean ± SEM. * *p* < 0.05, ** *p* < 0.01.

Spontaneous alternation showed an age-dependent pattern following prenatal VPA exposure. No significant treatment differences were detected at PND30 in either sex, whereas at PND70 spontaneous alternation was reduced in VPA300 males compared with saline controls (Figure 7A). No significant treatment differences were detected in females at either age (Figure 7B). Linear mixed-effects analysis, with litter included as a random effect and age as a repeated measure within individual offspring, showed a significant Treatment × Age interaction (F(2, 127.828) = 8.321, *p* < 0.001), whereas the main effects of treatment (F(2, 15.503) = 3.178, *p* = 0.070), sex (F(1, 117.129) = 0.439, *p* = 0.509), and age (F(1, 128.162) = 0.487, *p* = 0.486) were not significant. The Treatment × Sex (F(2, 116.336) = 1.780, *p* = 0.173), Sex × Age (F(1, 128.162) = 0.558, *p* = 0.456), and Treatment × Sex × Age (F(2,127.828) = 0.890, *p* = 0.413) interactions were also not significant.

Pairwise comparisons showed no significant treatment differences in males at PND30, whereas at PND70 spontaneous alternation was reduced in VPA300 males compared with saline controls (*p* = 0.007). VPA600 males did not differ significantly from saline. In females, no pairwise treatment comparisons reached significance after correction for multiple comparisons at either age.

To determine whether differences in spontaneous alternation could be associated with altered maze exploration, total arm entries were analyzed using the same mixed-effects approach. Total arm entries also varied with treatment and age. No significant treatment differences were detected in males at either age or in females at PND30, whereas VPA300 females made more arm entries than saline females at PND70 (Figure 6C,D).

Mixed-effects analysis showed a significant Treatment × Age interaction (F(2, 132.714) = 7.071, *p* = 0.001), whereas the main effects of treatment (F(2, 16.424) = 2.329, *p* = 0.129), sex (F(1, 118.523) = 0.309, *p* = 0.580), and age (F(1, 134.820) = 0.757, *p* = 0.386) were not significant. The Treatment × Sex (F(2, 117.272) = 2.068, *p* = 0.131), Sex × Age (F(1, 134.820) = 0.087, *p* = 0.768), and Treatment × Sex × Age (F(2, 132.714) = 0.155, *p* = 0.857) interactions were also not significant. Pairwise comparisons confirmed no significant treatment differences in males at either age or in females at PND30. At PND70, VPA300 females made more arm entries than saline females (*p* = 0.035).

Thus, Y-maze performance showed an age-dependent treatment pattern, with significant treatment differences emerging at PND70. The reduced spontaneous alternation observed in VPA300 males at PND70 was not accompanied by reduced arm-entry frequency, while the increased arm entries observed in VPA300 females at PND70 occurred without a significant change in spontaneous alternation. However, these findings did not establish direct VPA300–VPA600 differences or sex-specific treatment effects. Because this finding was limited to VPA300 males at PND70 and was not supported by a significant overall treatment effect or a significant Treatment × Sex × Age interaction, it should be interpreted cautiously and not as definitive evidence of a specific working-memory deficit.

In summary, prenatal VPA exposure produced neurodevelopmental effects that varied according to dose, sex, age, and behavioral domain. Early postnatal development was largely preserved, although alterations in the timing of selected developmental milestones were observed, most notably for cliff aversion following the 300 mg/kg dose. Total USV production was also largely preserved, whereas analysis of syllable composition revealed alterations in selected call types, including reduced Flat calls in VPA-exposed males at PND8. In contrast, later behavioral alterations varied according to dose, sex, age, and behavioral measure. The 600 mg/kg dose was associated with greater embryonic mortality, whereas behavioral alterations differed between the two doses according to age and behavioral domain. These findings indicate that among surviving offspring, behavioral outcomes differed between VPA300 and VPA600 across developmental stages and behavioral domains, without a consistent increase in severity at the higher dose. However, the substantially greater embryonic mortality associated with VPA600 should be considered when interpreting behavioral outcomes in the surviving offspring. A comparison of the effects of 300 and 600 mg/kg VPA is summarized in Table 3.

## 4. Discussion

The present study provides a longitudinal characterization of neurological development and behavioral outcomes following prenatal exposure to intermediate and high doses of valproate administered on day 12.5 of gestation. The higher dose was associated with substantially increased intrauterine mortality, with VPA600 differing significantly from saline, although the direct VPA300–VPA600 comparison was not significant. Among surviving offspring, we assessed early communication (ultrasonic vocalizations), neurodevelopmental milestones, and later behavioral functions, including locomotion, center exploration, sociability, and Y-maze performance. We found that prenatal VPA exposure did not produce a uniform increase in severity with higher dose. Instead, the effects varied between behavioral outcomes and changed with age, with sex also contributing to several of these patterns. Among the early developmental milestones, cliff aversion showed the clearest treatment effect. Acquisition was significantly delayed in VPA300 females compared with both saline and VPA600 females, providing direct evidence of a difference between the two VPA doses for this outcome. However, because the Treatment × Sex interaction was not significant, this finding should be interpreted as a within-female dose difference rather than a sex-specific effect. Total USV production was also largely preserved, whereas selected aspects of call composition were altered. Later behavioral changes involved both doses and varied according to behavioral measure, age, and sex. Thus, increasing VPA dose did not simply increase the severity of neurodevelopmental effects but was associated with different patterns of outcomes across developmental stages and behavioral domains (Table 3).

Previous studies in mice using single-dose, single-time-point designs report robust ASD-like phenotypes following prenatal VPA exposure [8,20,24,42,43,44,45,46]. The present findings extend these observations by demonstrating that behavioral alterations following prenatal VPA exposure vary across behavioral domains and developmental ages, with different patterns observed between the two doses and between sexes. However, it should be noted that the present study does not assess all behavioral domains relevant to ASD, such as repetitive behaviors [47]. Therefore, it was not intended to define a complete ASD-like phenotype but to characterize specific neurobehavioral alterations induced by prenatal VPA exposure.

The present study also demonstrated that sex contributed to the neurobehavioral consequences of prenatal VPA exposure, although the extent of this influence differed between behavioral measures. A significant Treatment × Sex interaction was observed for sociability, and locomotor activity showed a significant Treatment × Sex × Age interaction. In other outcomes, treatment effects were observed within one sex without a significant higher-order interaction, and these findings therefore should not be interpreted as evidence of a statistically established sex-specific effect.

Sex hormones play a central role in brain sexual differentiation by regulating neuronal proliferation, migration, synaptogenesis, and circuit maturation during prenatal and early postnatal development [48,49]. Sex differences are also evident in fetal antioxidant systems, including differences in SOD activity and lipid peroxidation [50], which may contribute to differential susceptibility to prenatal environmental insults [29,51], including sex-dependent responses to VPA exposure [11]. Such vulnerability appears to be region-specific rather than uniformly greater in either sex [52]. Importantly, prenatal oxidative stress has been associated with sex-dependent later neurobehavioral outcomes [53], and prenatal VPA exposure produced sex- and region-dependent alterations in brain glutathione together with distinct behavioral abnormalities in male and female offspring [54]. Together, these findings support the possibility that sex-dependent redox vulnerability during development may contribute to differences in later neurobehavioral outcomes.

In addition, astroglial maturation, synaptic remodeling, and corticolimbic circuit development follow sex-dependent developmental trajectories, which may create different windows of vulnerability across postnatal development [30,55,56,57]. Such differences in developmental pacing may help explain why prenatal VPA-induced alterations that are not evident in early sensorimotor functions become behaviorally apparent later, during adolescence and adulthood, as circuits underlying complex social behavior continue to mature and undergo refinement. Although these mechanisms were not directly investigated in the present study, they provide a possible developmental framework for the later-emerging and sex-related behavioral effects observed in our offspring.

USV production showed a pronounced developmental trajectory, increasing to a peak at PND8 and declining by PND14, but total call number was not significantly affected by prenatal VPA exposure in either sex. This differs from several prenatal VPA studies reporting reductions in USV emission following prenatal exposure [58,59]. However, analysis of call composition indicated that VPA affected selected components of the vocal repertoire. In particular, Flat calls were reduced in VPA-exposed males at PND8, whereas no corresponding treatment difference was detected in females. Because the Treatment × Sex × Age interaction was not significant, this finding should not be interpreted as evidence of a statistically established male-specific effect. Other less frequent syllable types also showed statistical interactions, but their low occurrence limits biological interpretation. Thus, prenatal VPA exposure did not produce a generalized suppression of vocalization but was associated with alterations in selected components of the developing vocal repertoire.

VPA-induced USV abnormalities vary with developmental age and the vocalization parameter examined. In ICR mice exposed to VPA at E12.5, Tsuji et al. [60] reported loss of the normal developmental peak in USVs and reduced call rates at PND11 in both sexes. Similarly, Agarwalla et al. [59] identified age- and sex-related alterations across different USV parameters, including vocal sequence structure. Differences in experimental procedures may also contribute to variation among studies. In previous VPA studies, isolation-induced USVs were recorded immediately after individual separation from the dam and littermates [59,60], whereas in the present study, pups were separated from the dam for 30 min while remaining with their littermates before individual USV recording. Because USVs can be influenced by separation conditions and temperature [61,62], this procedural difference may partly explain why we did not observe the reductions in total call number reported in some previous VPA studies. Pup body temperature was not monitored during the separation period, which should also be considered a possible limitation.

Early neurodevelopmental milestones were largely preserved following prenatal VPA exposure. Surface righting and negative geotaxis showed no significant overall treatment effects on acquisition timing after accounting for litter. In contrast, cliff aversion showed a significant treatment effect, with later acquisition following VPA300 exposure, whereas VPA600 did not differ significantly from saline. Pairwise differences were observed among females, although the Treatment × Sex interaction was not significant. Importantly, nearly all offspring ultimately acquired the developmental milestones within the assessment period. These findings suggest that prenatal VPA exposure can delay the acquisition of selected early neurodevelopmental milestones without producing a generalized or persistent impairment, consistent with previous mouse studies [63,64,65].

Early neurodevelopmental milestones, including surface righting, negative geotaxis, and cliff aversion, depend on the rapid maturation of brainstem and cerebellar circuits integrating vestibular, proprioceptive, tactile, and spinal motor pathways [37,66,67]. In rodents, this early postnatal period corresponds to late gestation in humans and is characterized by intense circuit formation and refinement [68,69]. Because these neural systems mature within a relatively narrow developmental window, even subtle prenatal disturbances may transiently delay sensorimotor development [69]. This concept is consistent with reports describing cerebellar abnormalities [65,70,71,72,73,74] and altered inhibitory signaling following prenatal VPA exposure [64,75,76].

In contrast, behavioral alterations were more evident at later ages, particularly in social behavior, and varied according to developmental age, behavioral domain, and sex. Importantly, these effects were not uniformly greater following the higher VPA dose. Previous prenatal VPA studies have similarly reported alterations in social behavior, exploratory activity, and cognitive performance during adolescence and adulthood [8,20,24,42,43,44,45,46], although the magnitude and sex distribution of these effects vary among studies.

Later behaviors, including anxiety-related behavior, sociability, and spatial working-memory related behaviors, depend on higher-order cortical and cortico-subcortical networks that continue to mature throughout postnatal development. Disruption of these circuits during prenatal life may therefore remain clinically silent until they reach functional maturity. This developmental pattern is also recognized in human neurodevelopmental disorders, in which abnormalities in early brain development are often observed clinically only during childhood or adolescence [77,78].

Experimental studies have demonstrated that prenatal VPA exposure alters cortical development, synaptic organization, excitation–inhibition balance, and microglial function [30,58,79,80,81,82]. Although these mechanisms were not examined in the present study, they may provide a possible explanation for why several behavioral abnormalities emerged only during adolescence and adulthood. The differing observed patterns between VPA300 and VPA600 further suggest that the behavioral consequences of prenatal VPA exposure may change as development progresses. Whether this reflects delayed maturation, developmental compensation, or other adaptive mechanisms remains unknown.

A key question arising from our findings is why the two VPA doses were associated with different developmental and behavioral outcome effects, and the higher dose did not consistently produce more severe outcomes. This apparent paradox likely reflects the involvement of multiple interacting mechanisms or may be related to brain maturation.

One possible explanation relates to the greater embryotoxicity associated with higher VPA doses. Higher VPA doses are known to cause greater embryonic loss [83,84], a finding that was also observed in the present study. The offspring surviving higher doses may represent a selected population that differs from those surviving after intermediate-dose exposure. Therefore, the absence of a consistently more severe behavioral phenotype in the VPA600 group should not be interpreted as evidence of a weaker overall neurodevelopmental effect of the higher dose, as some of the most vulnerable embryos may not have survived to postnatal behavioral assessment. Early behavioral alterations may be more readily detected after the intermediate dose, whereas higher doses may result in more variable behavioral outcomes among the surviving offspring. However, this possibility was not directly examined in the present study.

Previous studies also indicate that relatively small differences in VPA exposure can produce markedly different developmental outcomes. In ICR mice, an increase in VPA dose from 550 to 600 mg/kg during mid-gestation produced a marked shift from partial embryonic loss to near-complete embryonic lethality, illustrating the strong dependence of developmental toxicity on exposure level [85]. Similarly, high VPA doses (500–600 mg/kg) in E12.5 mouse models have been associated with both embryonic loss and neurobehavioral abnormalities among surviving offspring [8].

Podgorac et al. examined multiple prenatal VPA dosing regimens and reported that behavioral outcomes varied according to dose, sex, and behavioral measure, instead of a dose-related increase in severity with increasing dose [44,63]. Although their exposure paradigm differed from the present study, their findings are consistent with our observation that the two VPA doses were associated with different patterns of behavioral findings across developmental stages and behavioral domains. Together, these findings emphasize that the consequences of prenatal VPA exposure depend on the developmental endpoint examined and that prenatal exposure to higher doses should not be assumed to produce proportionally greater behavioral impairment among surviving offspring.

Strain background should also be considered when comparing the present findings with previous prenatal VPA studies. The present study was conducted in outbred ICR mice, whereas many studies of the prenatal VPA model have used inbred C57BL/6 mice. Different VPA doses have been used to induce neurodevelopmental alterations across these experimental models. Thus, 300 mg/kg VPA administered at E12.5 produced neurodevelopmental and behavioral alterations in ICR offspring [17,28], whereas studies using C57BL/6 mice commonly employ higher doses, including 500–600 mg/kg [19,20,31]. These differences suggest that genetic background may influence sensitivity to prenatal VPA and should be considered when interpreting dose-related effects across strains. However, the present study directly compared the two VPA doses within the same ICR background and under identical experimental conditions, thereby minimizing strain-related variability within the dose comparison.

An important strength of the present study is the direct comparison of two commonly used prenatal VPA doses within the same experimental design and under identical experimental conditions. Direct comparisons between these widely used prenatal VPA regimens are relatively uncommon, despite their frequent use in rodent models of neurodevelopmental disorders. The study employed a longitudinal design in which offspring were evaluated from early postnatal development through PND70 using complementary assessments of developmental milestones, ultrasonic vocalizations, locomotor activity, social behavior, and Y-maze performance. Whenever possible, the same offspring were assessed across developmental stages, allowing age-related changes to be examined within a longitudinal framework. Together, these features provide a comprehensive characterization of the developmental and behavioral consequences of prenatal exposure to two commonly used VPA doses. In addition, males and females were analyzed separately throughout the study, enabling evaluation of sex-related outcome patterns under standardized experimental conditions.

## 5. Limitations

This study also has several limitations that should be considered. A major limitation of this study is the relatively small number of independent litters, particularly in the VPA-treated groups in the later behavioral assessments. Although litter was included as a random effect, the limited number of litters may reduce the precision of estimated treatment effects and higher-order interactions. No formal a priori power calculation was performed, and the study included multiple developmental and behavioral outcomes without a prespecified primary endpoint. Multiplicity adjustment was performed within individual analytical domains rather than across all study endpoints; therefore, the findings should be considered exploratory, particularly those with *p*-values close to the significance threshold. Formal blinding to prenatal treatment allocation was not implemented. Although USV processing and later behavioral measurements were largely automated, manually assessed developmental milestones may have been susceptible to observer bias. Finally, maternal body weight and food intake were not monitored following VPA administration, limiting distinction between direct embryonic and indirect maternal effects.

## 6. Conclusions

Among surviving offspring, the examined outcomes did not show a consistent monotonic increase in behavioral severity from 300 to 600 mg/kg. The two commonly used VPA doses of 300 and 600 mg/kg were associated with partially different developmental and behavioral outcome patterns across postnatal development, although these patterns did not consistently represent statistically demonstrated differences between doses. The substantially greater embryonic mortality associated with 600 mg/kg complicates direct comparison of behavioral outcomes among surviving offspring and may have contributed to the absence of a consistently more severe behavioral phenotype at the higher dose. The emergence of behavioral alterations at later developmental stages despite relatively preserved early sensorimotor development further emphasizes the importance of developmental timing when evaluating the consequences of prenatal VPA exposure. Together, these findings highlight dose, developmental stage, sex, and survival as important considerations in the design and interpretation of prenatal VPA models of neurodevelopmental disorders. Future studies integrating behavioral outcomes with neuroanatomical, biochemical, and gene expression analyses are needed to identify the mechanisms underlying these differences.

## Figures and Tables

**Table 1:** Effect of Prenatal VPA treatment on embryonic mortality.

Outcome	Saline	VPA 300 mg/kg	VPA 600 mg/kg
Number of litters (n)	6	6	5
Total fetuses	73	75	61
Mean fetuses per litter	12.2	12.5	12.2
Viable fetuses	72	69	44
Non-viable fetuses	1	6	17
Pooled mortality, % *	0.95	8.5	33.7
Litter mortality, % (Mean + SEM)	1.5 ± 1.5	8.5 ± 4.184	32.58 ± 14.5

* Pooled fetal counts are presented for descriptive purposes only. Statistical analysis was performed using the mortality percentage of each individual litter as the experimental unit. Kruskal–Wallis: H = 8.934, exact *p* = 0.0059; Dunn’s adjusted comparisons: Saline vs. VPA300, *p* = 0.8309; Saline vs. VPA600, *p* = 0.0091; VPA300 vs. VPA600, *p* = 0.1611.

**Table 2:** Developmental milestone acquisition following prenatal VPA exposure.

Test	Sex	Treatment	n	PND4	PND5	PND6	PND7	PND8	PND9		Acquisition Day, EMM ± SE
Surface righting	Male	Saline	28	26/28 (92.9%)	26/28 (92.9%)	28/28 (100%)	28/28 (100%)	28/28 (100%)	28/28 (100%)		4.155 ± 0.301
		VPA 300 mg/kg	34	21/34 (61.8%)	26/34 (76.5%)	31/34 (91.2%)	33/34 (97.1%)	33/34 (97.1%)	34/34 (100%)		4.762 ± 0.285
		VPA 600 mg/kg	27	14/27 (51.9%)	22/27 (81.5%)	24/27 (88.9%)	26/27 (96.3%)	27/27 (100%)	27/27 (100%)		5.113 ± 0.302
	Female	Saline	34	28/34 (82.4%)	31/34 (91.2%)	33/34 (97.1%)	33/34 (97.1%)	34/34 (100%)	34/34 (100%)		4.397 ± 0.283
		VPA 300 mg/kg	41	24/41 (58.5%)	29/41 (70.7%)	36/41 (87.8%)	40/41 (97.6%)	41/41 (100%)	41/41 (100%)		4.818 ± 0.266
		VPA 600 mg/kg	53	32/53 (60.4%)	40/53 (75.5%)	44/53 (83.0%)	49/53 (92.5%)	51/53 (96.2%)	53/53 (100%)		5.107 ± 0.265
Negative geotaxis	Male	Saline	28	23/28 (82.1%)	23/28 (82.1%)	28/28 (100%)	28/28 (100%)	28/28 (100%)	28/28 (100%)		4.342 ± 0.171
		VPA 300 mg/kg	34	27/34 (79.4%)	29/34 (85.3%)	32/34 (94.1%)	32/34 (94.1%)	33/34 (97.1%)	34/34 (100%)		4.407 ± 0.159
		VPA 600 mg/kg	27	25/27 (92.6%)	25/27 (92.6%)	25/27 (92.6%)	26/27 (96.3%)	27/27 (100%)	27/27 (100%)		4.298 ± 0.173
	Female	Saline	34	32/34 (94.1%)	34/34 (100%)	34/34 (100%)	34/34 (100%)	34/34 (100%)	34/34 (100%)		4.041 ± 0.159
		VPA 300 mg/kg	39	28/39 (71.8%)	35/39 (89.7%)	38/39 (97.4%)	39/39 (100%)	39/39 (100%)	39/39 (100%)		4.430 ± 0.152
		VPA 600 mg/kg	35	32/35 (91.4%)	34/35 (97.1%)	34/35 (97.1%)	35/35 (100%)	35/35 (100%)	35/35 (100%)		4.137 ± 0.162
Cliff aversion	Male	Saline	28	27/28 (96.4%)	28/28 (100%)	28/28 (100%)	28/28 (100%)	28/28 (100%)	28/28 (100%)		4.035 ± 0.206
		VPA 300 mg/kg	34	29/34 (85.3%)	30/34 (88.2%)	31/34 (91.2%)	31/34 (91.2%)	33/34 (97.1%)	34/34 (100%)		4.371 ± 0.195
		VPA 600 mg/kg	27	27/27 (100%)	27/27 (100%)	27/27 (100%)	27/27 (100%)	27/27 (100%)	27/27 (100%)		4.049 ± 0.211
	Female	Saline	34	32/34 (94.1%)	33/34 (97.1%)	34/34 (100%)	34/34 (100%)	34/34 (100%)	34/34 (100%)		4.082 ± 0.188
		VPA 300 mg/kg	40	31/40 (77.5%)	32/40 (80.0%)	33/40 (82.5%)	33/40 (82.5%)	36/40 (90.0%)	37/40 (92.5%)		4.864 ± 0.177
		VPA 600 mg/kg	53	50/53 (94.3%)	52/53 (98.1%)	52/53 (98.1%)	53/53 (100%)	53/53 (100%)	53/53 (100%)		4.147 ± 0.159
Mixed-effects model of acquisition day						Significant Bonferroni-adjusted pairwise comparisons					
Test/effect		F	df1	df2	*p*	Test	Sex	Comparison		Adjusted *p*	
Surface righting:Treatment		2.60	2.0	21.02	0.098	Cliff aversion	Female	Saline vs. VPA 300 mg/kg		0.011	
Surface righting: Sex		0.49	1.0	198.26	0.49						
Surface righting:Treatment × Sex		0.27	2.0	198.24	0.76			VPA 300 vs. VPA 600 mg/kg		0.009	
Negative geotaxis:Treatment		0.89	2.0	25.35	0.42						
Negative geotaxis:Sex		1.89	1.0	189.20	0.17	Complex samples Cox proportional-hazards regression					
Negative geotaxis: Treatment × Sex		0.84	2.0	186.04	0.43	Cliff aversion	Wald F(2, 19) = 4.81, *p* = 0.020	VPA300 vs SAL: HR = 0.611, 95% CI 0.422–0.885, *p* = 0.012			
Cliff aversion: Treatment		4.86	2.0	22.15	0.018						
Cliff aversion: Sex		2.23	1.0	206.98	0.14			VPA600 vs SAL: HR = 1.049, 95% CI 0.871–1.262, *p* = 0.600			
Cliff aversion:Treatment × Sex		0.99	2.0	207.79	0.37						

Cumulative responder percentages are presented descriptively. Acquisition day is reported as estimated marginal mean (EMM) ± SE from a linear mixed-effects model with Treatment, Sex, and Treatment × Sex as fixed effects and litter as a random effect, except where day-specific responder status was additionally evaluated using litter-adjusted binomial GEE, as reported in the Results. For cliff aversion, one VPA300 female did not acquire the response by PND12 and was excluded from the acquisition-day model. Significant *p*-values are highlighted in red.

**Table 3:** Comparison of prenatal VPA exposure effects across developmental and behavioral domains.

Domain	VPA300 mg/kg	VPA600 mg/kg	Statistical Interpretation of Dose Patterns
	Effect	Effect	
Embryonic mortality	Mild Increase, not significantly different from saline	Marked increase, significantly higher mortality than saline	Greater observed mortality with VPA600; direct VPA300–VPA600 comparison not significant
Total USV calls	No significant effect	No significant effect	No significant treatment differences detected.
USVs—Flat calls	Reduced at PND8 in males	Reduced at PND8 in males	Reduced Flat calls were observed with both VPA doses at PND8 in males; no direct dose difference was demonstrated
Body weight	No significant effect	No significant effect	Overall postnatal growth was preserved; transient VPA300–VPA600 differences occurred in males at selected ages.
Surface righting	No significant effect	No significant effect	No significant treatment differences detected.
Negative geotaxis	No significant effect	No significant effect	No significant treatment differences detected.
Cliff aversion	Significant treatment effect; Delayed acquisition	No significant difference from saline	VPA300 showed later acquisition than both saline and VPA600 in females
Open field locomotion	Decreased locomotion at PND70 in females	Decreased locomotion at PND30 in females	Age- and sex-dependent treatment patterns: VPA600 differed from saline at PND30 and VPA300 differed from saline at PND70 in females; direct dose differences were not established at PND70
Open field center time	No significant effect	Reduced center exploration at PND30 and PND70 in females	At PND30, VPA600 females showed lower center exploration than both saline and VPA300; at PND70, VPA600 differed from saline, but no significant VPA300–VPA600 difference was detected.
3ChT Sociability index	Reduced at PND30 and PND70 in both male and female	Reduced at PND30 and PND70 in males; reduced at PND70 in females	Sociability showed treatment-, sex-, and age-related variation; no significant VPA300–VPA600 difference was detected at either age.
Stranger preference	Absent preference in females at PND30; reversed in both sexes at PND70	Preserved preference at PND30; absent in males at PND70	Stranger-preference patterns varied with treatment and age; disruption at PND70 was observed in VPA300 offspring, but a direct VPA300–VPA600 difference was not detected.
Y-maze spontaneous alternation	Reduced spontaneous alternation at PND70 in males	No significant effect	VPA300 males showed reduced spontaneous alternation vs. saline at PND70; no direct VPA300–VPA600 difference was demonstrated
Y-maze arm entries	Increased arm entries at PND70 in females	No significant effect	VPA300 females showed increased arm entries vs. saline at PND70; no direct VPA300–VPA600 difference was demonstrated.

## Data Availability

Data will be made available on request.

## Supplementary Materials

The following supporting information can be downloaded at: https://www.mdpi.com/article/10.3390/neurosci7050112/s1, Figure S1: Experimental Cohort and overlap of litters included in the present manuscript; Table S1: Statistical summary of developmental and behavioral outcomes.

## Abbreviations

The following abbreviations are used in this manuscript:

ASD	Autism Spectrum Disorder
CA	Cliff aversion
E12.5	Embryonic day 12.5
NG	Negative geotaxis
NTDs	Neural Tube Defects
PND	Postnatal day
SR	Surface righting
USVs	Ultrasonic vocalizations
VPA	Valproic acid
