# Supplementary material for: Direct Comparison of Two Commonly Used Prenatal Valproate Doses Identifies Different Sex- and Age-Related Neurobehavioral Outcomes Across Development
Source: NeuroSci. 2026 Oct 19;7(5):112. doi: 10.3390/neurosci7050112 (PMC13708558; doi:10.3390/neurosci7050112)
Supplement: Supplementary file 1 [file neurosci-07-00112-s001.zip › Supplementary Figure S1.pdf]

Supplementary Figure S1. Experimental Cohort and overlap of litters included in the present manuscript.

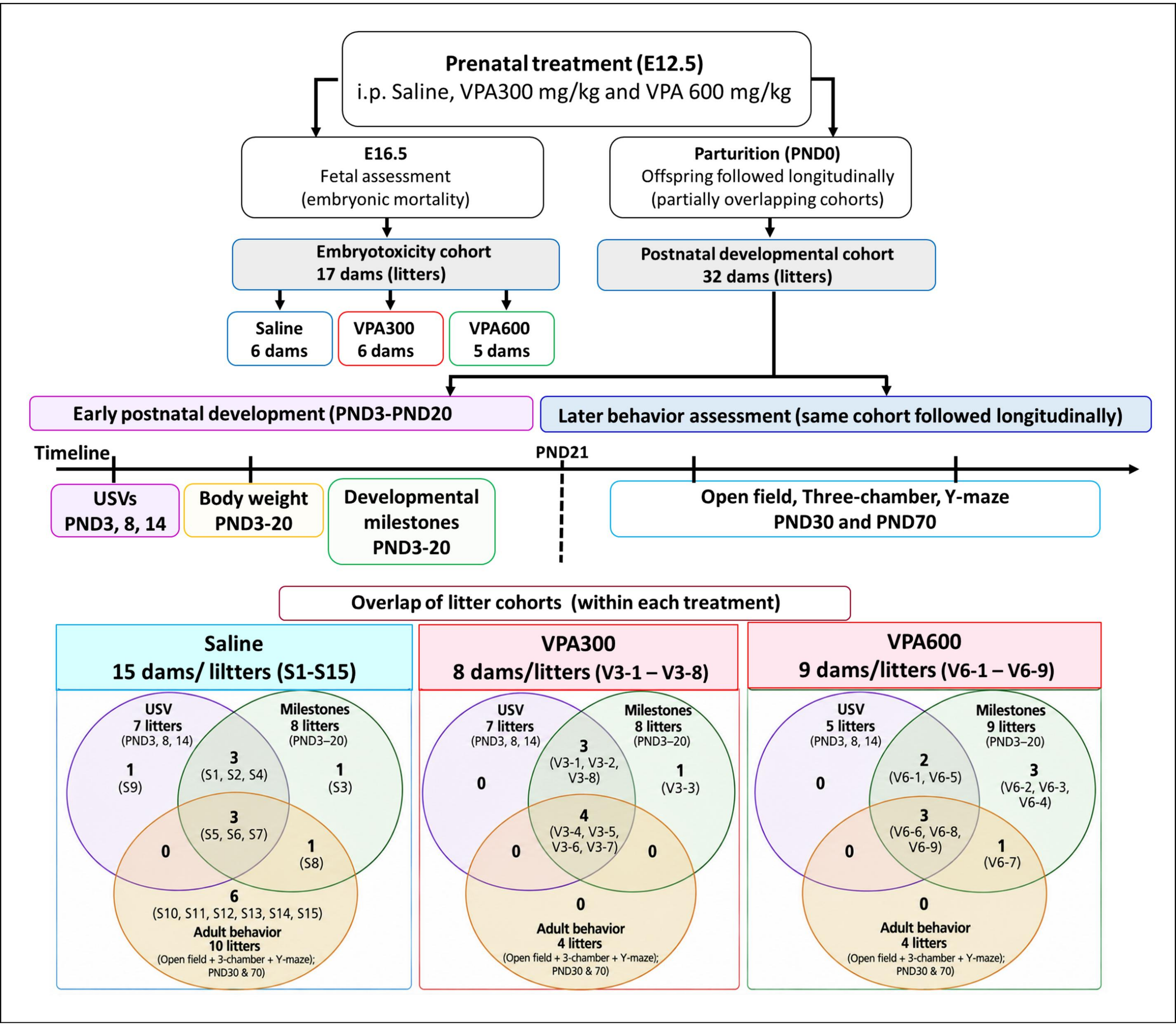

Supplementary Figure S1. Cohort allocation, longitudinal follow-up, and overlap of litter cohorts included in the present manuscript. Pregnant ICR dams were assigned to saline, VPA300, or VPA600 treatment groups at E12.5. The study included an embryotoxicity cohort assessing embryonic mortality at E16.5 and a postnatal cohort assessed from birth through adulthood. Early postnatal assessments included ultrasonic vocalizations (PND3, PND8, and PND14), body-weight monitoring (PND3-20), and developmental milestone testing (PND3-20). Behavioral assessments (open field, three-chamber social interaction, and Y-maze) were performed longitudinally in the same offspring at PND30 and PND70. Venn diagrams show the overlap of litter cohorts (treatment-specific serial dam IDs) contributing to USV, developmental milestone, and adult behavioral assessments within each treatment group. Numbers indicate the number of litters in each exclusive or overlapping region, with litter IDs shown in parentheses. Developmental milestone assessments used largely overlapping litter cohorts, with only minor endpoint-specific differences in the number of contributing litters. Differences in sample size among outcomes therefore reflect intentional cohort allocation rather than sequential attrition. Additional offspring from some litters were allocated to parallel biochemical and molecular studies not included in the present manuscript. Exact numbers of animals and contributing litters for each analysis are provided in Supplementary Table S1.
